# Behavior-Based Cleaning for Unreliable RFID Data Sets

**DOI:** 10.3390/s120810196

**Published:** 2012-07-30

**Authors:** Hua Fan, Quanyuan Wu, Yisong Lin

**Affiliations:** 1 School of Computer Science, National University of Defense Technology, Changsha 410073, China; E-Mail: quanyuan.wu@gmail.com; 2 Logistics Science Research Institute of GLD, Beijing 100071, China; E-Mail: linyisong@live.cn

**Keywords:** RFID technology, miss reading, data cleaning, movement behavior, kinematic characteristics

## Abstract

Radio Frequency IDentification (RFID) technology promises to revolutionize the way we track items and assets, but in RFID systems, missreading is a common phenomenon and it poses an enormous challenge to RFID data management, so accurate data cleaning becomes an essential task for the successful deployment of systems. In this paper, we present the design and development of a RFID data cleaning system, the first declarative, behavior-based unreliable RFID data smoothing system. We take advantage of kinematic characteristics of tags to assist in RFID data cleaning. In order to establish the conversion relationship between RFID data and kinematic parameters of the tags, we propose a movement behavior detection model. Moreover, a Reverse Order Filling Mechanism is proposed to ensure a more complete access to get the movement behavior characteristics of tag. Finally, we validate our solution with a common RFID application and demonstrate the advantages of our approach through extensive simulations.

## 1. Introduction

Radio Frequency Identification (RFID) is an electronic tagging technology that allows objects, places, or persons to be automatically identified at a distance without a direct line-of-sight, using an electromagnetic challenge/response exchange [1,2]. RFID offers a possible alternative to barcodes, and has emerged as a key technology for a wide-range of applications, including supply chain, retail stores, and asset management [3]. However, the widespread adoption of RFID technology is limited for the unreliability of the data streams produced by RFID readers [4,5]. RFID data cleaning is therefore widely considered as a principal challenge and has been an important research topic in the last few years [6–8].

Despite the improvement of the accuracy of RFID readers, there are still erroneous readings such as missed readings and ghost readings, due to interference, inappropriate placement of tags, temporary or permanent malfunction of some components.

The goal of RFID data cleaning is to eliminate the erroneous readings, especially to reduce or eliminate dropped readings. In this paper, we propose an innovative approach of cleaning RFID raw data Behavior-Based Smoothing for unreliable RFID data (BBS). Unlike conventional techniques, BBS relays primarily on the movement behavior of tags to fill the RFID data. Our biggest obstacle is how to obtain movement behavior characteristics of tags. To address this problem, a movement behavior detection model is proposed so that we can get the results by analyzing existing uncertain data of the corresponding tags. The contributions of this study are as follows:

- A movement behavior detection model. By counting the frequency of tags read in each cycle, we can get the read rate of tags and analyze kinematic characteristics of the tags according to changes of the read rate sequences, and ultimately to assist in RFID data cleaning.
- Reverse Order Filling Mechanism (ROFM). Based on the detection model, we design and implement a reversible RFID data filter. When we detect the data has not been filled completely, ROFM will be started to fill the data again in reverse order. The mechanism can ensure a more complete access to get the movement behavior characteristics of tags, and thus significantly improve the accuracy of data cleaning without scanning all the data twice.
- Improve the positioning accuracy of the RFID reader. Traditional RFID positioning system can only provide the Boolean result such as the condition whether the tag is in the read range of the reader at the time. But BBS can also get the distance between the tag and the reader, and even the velocity of tags.
- Evaluate the effect of BBS. We design several groups of contrast experiments on the data sets include measured data and simulation data. The results show that under all conditions with different missing rates, obviously, the precision of BBS is better than that of sliding-window cleaning.

The rest of this paper is organized as follows: we discuss the related work in Section 2. Section 3 defines the Object Movement Detection model and introduces our RFID data cleansing mechanism and arithmetic. An empirical evaluation of our solution is reported in Section 4. Finally, Section 5 concludes the paper.

## 2. Related Work

RFID technology has posed many challenges to database management systems, such as the requirements of supporting big volume data [9–11], handing new types of queries [11], event processing and data cleaning [5,12–16].

Many systems have been developed to manage uncertainty data. RFID data management, is one of the most important applications that drives the recent surge of interest in managing incomplete and uncertain data, which has been studied extensively. Valentine *et al.* [8] presented an adaptive sliding-window based approach WSTD for reducing false negative reads in RFID data streams. Rao *et al.* [13] presented a deferred approach for detecting and correcting RFID data anomalies by utilizing declarative sequenced-based rules. Chen *et al.* [14] proposed a Bayesian inference based approach, which takes full advantage of data redundancy, for cleaning RFID raw data. Gonzalez *et al.* [15] proposed a cleaning framework that takes an RFID data set and a collection of cleaning methods, with associated costs, and induces a cleaning plan that optimizes the overall accuracy adjusted cleaning costs by determining the conditions under which inexpensive methods are appropriates, and those when more expensive methods are absolutely necessary.

The work in [5,12] is the most relevant research to this paper. Jeffery *et al.* [5,12] proposed an adaptive smoothing filter SMURF for RFID data cleaning. SMURF focuses on a sliding-window aggregate that interpolates for lost readings. SMURF models the unreliability of RFID readings by taking RFID streams as a statistical sample of physical tags, and exploits techniques in sampling theory to drive its cleaning processes. But it is mainly applied to the circumstances that the movement of tags is infrequent, and is not effective in the case that tags move frequently.

## 3. Unreliable RFID Data Cleaning

### 3.1. A Movement Behavior Detection Model

The key for a movement behavior-based smoothing filter lies in how to establish the conversion relationship between read rate sequences and kinematic parameters of tags to assist in RFID data cleaning. To do so, we proposed a movement behavior detection model.

The process of tag passing through the reader's read range follows the laws of kinematics. The change of kinematic parameters such as displacement and velocity which possess an important feature is continuous, not transitional, so if the location (which mainly refers to the distance between tag and reader) and the relative velocity of tag at the time can be obtained through the original data, we can speculate the parameters of the tag at the missed reading time by these parameters and their trends, and further assist in data cleaning and improve its accuracy. BBS uses this approach, for example, using existing tag data to analyze and get the location *p*_1_ and the velocity *v*_1_ of the tag at the time *t*_1_, which can help approximately inferring to the relative location of the tag at the time *t*_1_ + *T* (*T* refers to a short period of time). Finally, by mapping the location information back to the RFID data, we can fill the missed RFID data. Therefore, through these kinematic parameters BBS can obtain whether the tag is in the detection range at the time, and further give its specific location.

**Figure 1.**
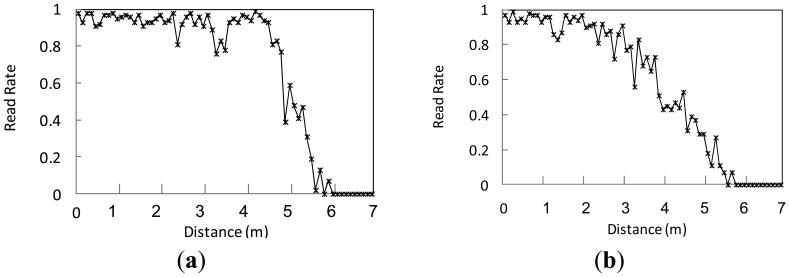
Read rate of tags in different conditions. (**a**) Quiet condition; (**b**) Noisy condition.

Adopting the statistical methods similar to SMURF, each epoch is viewed as an independent Bernoulli trial with success probability *p_i_* [12]. An epoch may be specified as a number of interrogation cycles or a unit of time. A typical epoch range is 0.2–0.25 seconds [5]. For each epoch, the reader keeps track of all the tags that have been identified, and additional information such as the number of interrogation responses for each tag and the last time the tag was read. Assuming, there are *n* interrogation cycles in an epoch, the number that *tag_i_* is monitored is *m_i_*. We can get the read rate of *tag_i_* at the moment by *p_i_* = *m_i_/n*. In the process of passing through the reader's read range, tags will be continuously scanned. Also in the whole process, the read rate of tag is not constant but constantly changing with the distance between the tag and reader. Besides, some researchers have proved by experiments that in the reader's detection region there is a linear relationship between read rate *p* and distance *s* [12]. For specific readers, the detection range *S* is a constant. To confirm this conclusion, we have carried out similar experiments and the conclusion is shown in Figure 1. The quiet condition means an ideal working environment of RFID devices with only a few interferences, while the noisy condition means a work environment with more interferences.

**Figure 2.**
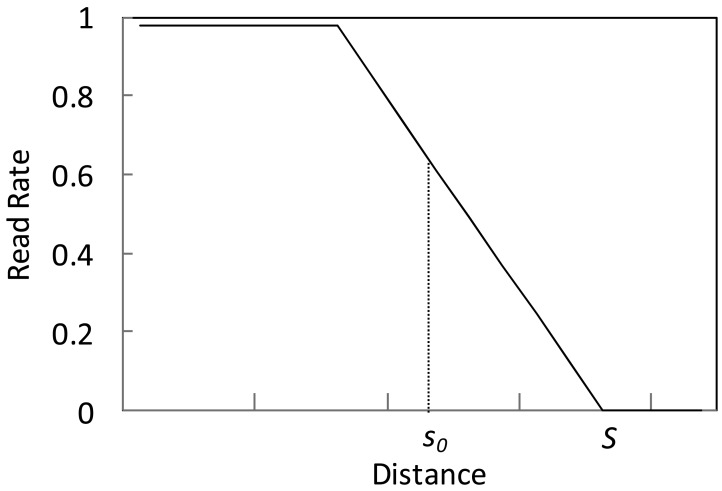
Relationship between read rate and distance.

By further abstraction of the conclusions above we get the relationship between read rate *p* and distance *s* in Figure 2. Obviously, the distance *s* between tag and reader and the read rate *p* follow the relation as:

(1) $$p=\{\begin{array}{ll} 0 & ks+b<0\\ ks+b & 0\leq ks+b\leq 1\\ 1 & ks+b>1 \end{array}$$

where, *b* = −*kS*, and *k* is the slope of the line, so above equation can be further written as:

(2) $$p=\{\begin{array}{ll} 0 & s>S\\ k(s-S) & 0\leq k(s-S)\leq 1\\ 1 & k(s-S)>1 \end{array}$$

### 3.2. Behavior-Based Smoothing for Unreliable RFID Data

In this section, how to use the model to fill the missed RFID data will be discussed. In our model, epoch is the basic unit of RFID data streams. Our mission is to fill in the missed epoch information. The information of RFID data stream that we get includes tag ID, the number of interrogation responses for each tag in an epoch and the time of the epoch, in the form of (*tag ID, Response number, time*). Let us analyze Equation (2). The read rate *p* can be calculated through *Response number*, and the detection range *S* is a constant, but the distance *s* can't be calculated directly. In practice, the detection region of each reader is generally not very large, ranging from a few meters to tens of meters. Therefore, the movement through the detection region for persons, vehicles and goods on the conveyor belt and other tagged items can be approximately considered as uniform linear motion or a combination of several successive uniform linear motions. In addition, even if the velocity and direction of the objects has obviously changed in this process, we can also break down their movement, and approximately consider each short process as uniform linear motion. Well known, the speed *v* of uniform linear motion satisfies the equation Δ*s* = *v*Δ*t*. And if we consider *s_0_* is the original distance of the tag, and Equation (2) can be further written as:

(3) $$p=\{\begin{array}{ll} 0 & K\Delta t+B<0\\ K\Delta t+B & 0\leq K\Delta t+B\leq 1\\ 1 & K\Delta t+B>1 \end{array}$$

where *K* = ± *kv* (It take the negative sign when the value of *p* increases, otherwise take the positive sign), and *B* = *k(s_0_−S)*.

In practice, readers are usually interfered by the surroundings including the signal reflection and obstruction or sudden current gain, *etc.*, so the read rate that is calculated by *Response number* will be unstable. The results from directly treating the raw data may differ from the actual movement characteristics, so we use *a* weighted moving average of order *n* to smooth the initial read rate sequences. The process of replacing the read rate sequences by its moving average eliminates unwanted fluctuations. Furthermore, the influence of extreme values can be reduced by employing a weighted moving average with appropriate weights to get more realistic movement features of items to be monitored. The calculation is as follows:

(4) $$p_{i}=\frac{w_{0}p_{i-\frac{n-1}{2}}^{\prime }+w_{0}p_{i-\frac{n-1}{2}+1}^{\prime }+\cdots +w_{1}p_{i}^{\prime }+\cdots +w_{0}p_{i+\frac{n-1}{2}}^{\prime }}{(\overset{i+\frac{n-1}{2}}{\underset{j=i-\frac{n-1}{2}}{\text{∑}}}\left\lceil p_{j}^{\prime }\right\rceil -1)w_{0}+w_{1}}$$

where *w*_1_ and *w*_0_ are the weights of read rate of current epoch and other epochs respectively.

In the above treatment, we only discuss such epoch whose read rate $p_{i}^{\prime }\neq 0$. When the read rate of the epoch is $p_{i}^{\prime }=0$, there are two possibilities: the tag is indeed outside the detection range or miss reading occurs to the tag, *i.e.*, the tag is in the detection range but not captured for interference factors. It is necessary for accurate data cleaning to distinguish these two cases clearly. We should analyze its movement feature in the adjacent time. The movement of tags is approximately uniform linear motion and satisfies Equation (3), so we can calculate the read rate *p_i_* of the tag by the value of *K* and the read rate *p_ia_* in the adjacent time, to further determine it is a true value or a missed reading. In order to solve the coefficient *K*, we denote *epoch_j_* = {*t_j_, p_j_*}, where *t_j_* and *p_j_* are the time and read rate of *epoch_j_* respectively, and a training set *TS* = {*epoch_i_*_+_*_l_* | *p_i-l_* ≠ 0, −*m* ≤ *l* ≤ *m*}, where the upper limit of |*TS*| is 2*m* + 1. So the coefficient *K* can be solved by the method of *least squares* on the trainings set of *TS*, which estimates the best-fitting straight line as the one that minimizes the error between the actual data and the estimate of the line:

(5) $$K_{i}=\frac{\underset{{\text{epoch}}_{j}\in TS}{\text{∑}}\left(p_{j}-\bar{p})(t_{j}-\bar{t}\right)}{\underset{{\text{epoch}}_{j}\in TS}{\text{∑}}{\left(p_{j}-\bar{p}\right)}^{2}}$$

(6) $$B_{i}=\bar{t}-K_{i}\bar{p}$$

where, $\bar{p}=\frac{\underset{{\text{epoch}}_{j}\in TS}{\text{∑}}p_{j}}{\left|TS\right|},\;\bar{t}=\frac{\underset{{\text{epoch}}_{j}\in TS}{\text{∑}}t_{j}}{\left|TS\right|}$.

### 3.3. Reverse Order Filling Mechanism (ROFM)

**Figure 3.**
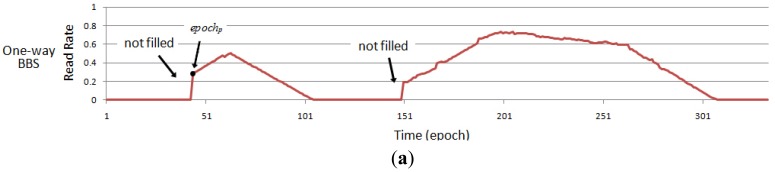

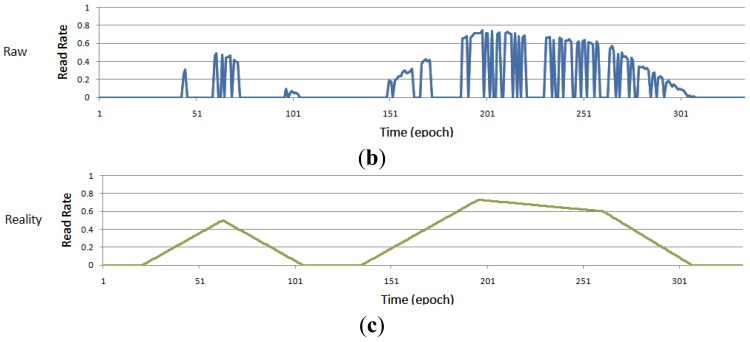
The cleaning result of one-way BBS. (**a**) one-way BBS; (**b**) raw; (**c**) reality.

In the data stream processing, data are normally processed in order. However, if the RFID data stream corresponding to a tag is filled in chronological order by the above-mentioned method, it is easy to bring the problem of miss filling, as shown in Figure 3(a). We analyze the read rate of a tag in one time period in detail in Figure 3. Figure 3(c) indicates the read rate of the tag without miss readings and Figure 3(b) shows the raw read rate that the reader actually read. For an *epoch_p_* in Figure 3(a), if the corresponding coefficient *K_p_* > 0 and the data before the time *t_p_* has been miss read for a long period of time, the data before a period of *t_p_* will not be filled because the RFID data stream are processed in order. A simple solution is to process the RFID data stream twice, forward and backward. However, this will add a lot of computational overhead. To solve this problem, we introduce a Reverse Order Filling Mechanism. As soon as we detect the situation mentioned above occurs, the read rate of the corresponding data stream is to be refilled in the reverse direction from *epoch_p_*_+_*_T_*. Until the original read rate *p_i_* ≠ 0 or the filling value of read rate *p_f_* = 0 the reverse filling mechanism will not be terminated. And the rest of data will be processed after that. So we only need a twice process to the corresponding data rather than all data, which ensures the completeness of RFID data cleaning, but also does not add too much computational overhead. Algorithm 1 shows a pseudo-code description of BBS cleaning algorithm.

**Algorithm 1.** A pseudo-code description of BBS cleaning algorithm.
**Algorithm BBS**
**Require:**
*Tags* = *set of all observed tag Ids*    *TS* = *the trainings set, the upper limit of* |*TS*| *is 2m*+*1***for** (*tag* in *Tags*) **do** **while** (*GetNextEpoch*()) **do**  *p_i_*=0  **if** (
$p_{i}^{\prime }!=0$) **then**   *p_i_*←*Smoothing*(*w*_0_, *w*_1_, *n*)// Equation 4   *K*←*CurrentSlope* (*TS*)// Equation 5   **if** (*p_i_*>*p_c_* && *K*>0 && *p*_*i*-1_==0) **then**   // check whether it is necessary to switch on ROFM or not    *t*_0_=*t_i_*    *GetEpoch*(*t_i_*+*T*)    **while** (*t_i_*≥*t*_0_ ‖ (*t_i_*<*t*_0_ && *p_i_*==0 && *Readrate*(*K, B, p_ia_, t_ia_, t_i_*)>0)) **do**     **if** (*p_i_*==0) **then**      *p_i_*←*Readrate*(*K, B, p_ia_, t_ia_, t_i_*) // Equation 3     **end if**    **end while**    *GetEpoch*(*t*_0_+*T*)   **end if**  **else if** (*p*_*i*-1_!=0) **then**   *K*←*CurrentSlope* (*TS*)// Equation 5   *p_i_*←*Readrate*(*K, B, p_ia_, t_ia_, t_i_*) // Equation 3  **else if** (*p*_*i*-1_==0) **then**   *p_i_*←*Readrate*(*K, B, p_ia_, t_ia_, t_i_*) // Equation 3  **end if** **end while****end for**


## 4. Experimental Evaluation

In this section, we present an analysis of the performance of BBS on several data sets and compare its accuracy with other cleaning methods. All the experiments were conducted on an Intel (R) Core (TM) 2 Duo CPU T9550 @ 2.66 GHz 2.67 GHz System with 2 GB of RAM. Our data include both the real collected data and simulation data. The laboratory equipments used for collecting data include Invengo XCRF-860 RFID UHF reader with 902–928 MHz frequency range, Invengo XCAF-12L antenna and XCTF-8101A tag. The simulation data for our experiments were generated by a synthetic RFID data generator that simulates the operation of RFID readers under a wide variety of conditions. We simulate various movements of tags with different missing rates. The missing rate means the probability that missed reading happens.

### 4.1. Accuracy Comparison

**Figure 4.**
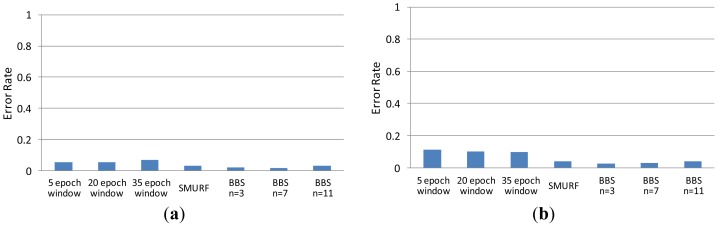

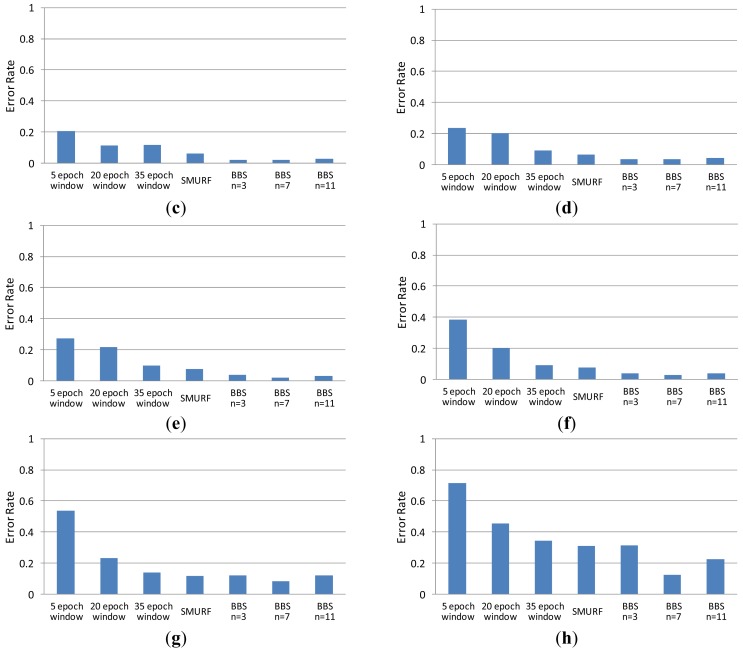
Accuracy comparison under different missing rates. (**a**) missing rate = 10%; (**b**) missing rate = 20%; (**c**) missing rate = 30%; (**d**) missing rate = 40%; (**e**) missing rate = 50%; (**f**) missing rate = 60%; (**g**) missing rate = 70%; (**h**) missing rate = 80%.

In the experiment, we compare the accuracy of data filled by BBS (with *n* = 3, *n* = 7 and *n* = 11, respectively), SMURF, and sliding-windows methods (with different window size: 5 epoch, 20 epoch and 35 epoch) under different missing rate (from 10% to 80%). The other experimental parameters of BBS are set as follows: *m* = 7, *w*_0_ = 1 and *w*_1_ = 2. We clean the same raw data with different methods. Comparing the corresponding cleaning result with real data, we can get the error rate of each method. As shown in Figure 4, the error rate of BBS is lower than that of sliding windows methods in all cases. We found that the choice of the parameter *n* will have some impact on the experimental results when the missing rate is greater than 70%. Therefore, in practical applications, for optimal cleaning results we should set parameters *n, m, w*_0_ and *w*_1_ with appropriate values in accordance with the actual needs. Usually, the more unstable the read rate sequence, the larger the value of *n* should be set; the higher the missing rate, the larger the value of *m* should be set.

**Figure 5.**
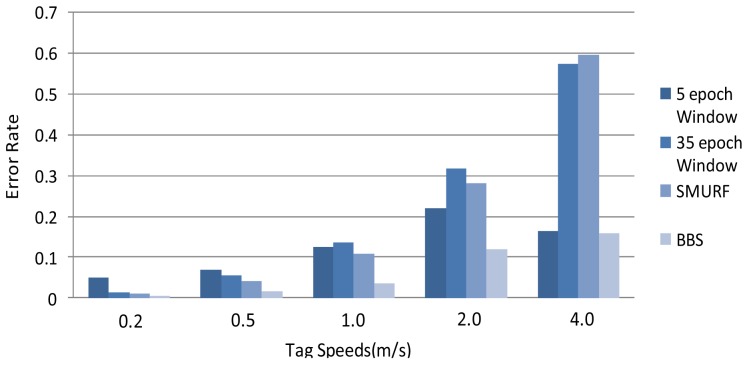
Accuracy comparison under different tag speeds.

We compare the accuracy of data filled by different methods under different tag speeds. The error rates obtained are used to compare the accuracy of methods where lower error rate means higher accuracy. As shown in Figure 5, the results of BBS are obviously superior to all other methods, especially when the speeds of tags are higher than 1.0 m/s.

**Figure 6.**
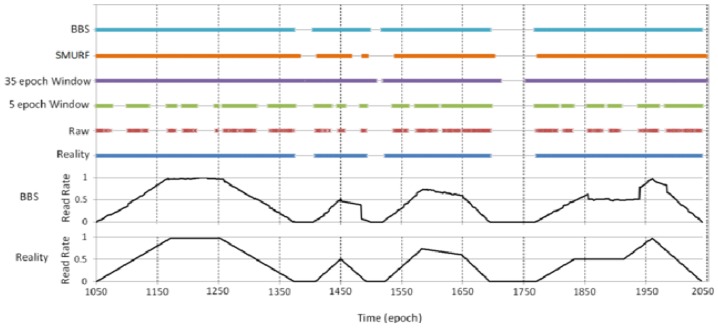
Analysis in detail.

Furthermore, we analyze the case in one time period in detail (missing rate = 50%, and the length of time is 1,000 epochs). As shown in Figure 6, Reality refers to readings that would have been produced by a perfect reader without missreadings. Raw means the raw data that the reader actually read and while the others refer to the data filled with four kinds of data cleaning methods (BBS, SMURF, 5 epoch sliding-window, and 35 epoch sliding-window). The bold horizontal lines indicate the tag is present/read, and vice versa. The line at the bottom of Figure 6 is the real data of read rate without miss reading, and another line above it is the estimate of read rate by BBS (*n* = 7, *w*_0_ = 1 and *w*_1_ = 2). Obviously, compared with sliding window methods, BBS greatly improved the accuracy of RFID data cleaning. In particular, our BBS method not only accurately draws whether the tag is in the read range of the reader, but also can give the read rate of each epoch. So BBS make it possible to get a more exact position of the tag.

### 4.2. Performance Comparison

**Figure 7.**
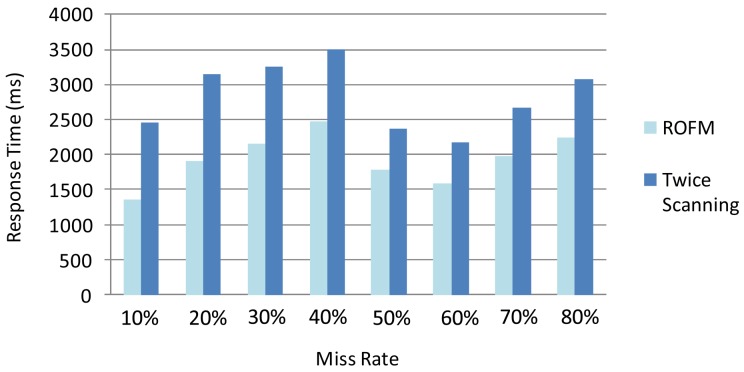
Performance comparison.

To verify the validity and necessity of Reverse Order Filling Mechanism, we design the following experiment. We focus on readings produced from a single tag with different missing rates from 10% to 80% in 10,000 epochs. We process 8 sets of data by two different methods, the Reverse Order Filling Mechanism method and the Twice Scanning Method, and determine their performance by comparing the response times. As show in Figure 7, the Reverse Order Filling Mechanism only needs a twice cleaning process to the corresponding data while the Twice Scanning Method needs the process to all data. Therefore, the former is obviously superior to the latter in the efficiency of processing data.

## 5. Conclusions

Accurate data cleaning is an essential task for the successful deployment of RFID systems. In this paper, we have proposed a behavior-based unreliable RFID data smoothing system BBS, which can take advantage of kinematic characteristics of tags to assist in RFID data cleaning. A movement behavior detection model is proposed to establish the conversion relationship between RFID data and kinematic parameters of the tags. Then we reduce the influence of extreme values and other unwanted fluctuations by employing a weighted moving average of order *n*. Moreover, Reverse Order Filling Mechanism (ROFM) is proposed for BBS to ensure a more complete access to get the movement behavior characteristics of tag. Finally, we validate our solution with a common RFID application and demonstrate the advantages of our approach through extensive simulations.

## References

[1] Evan W., Leilani B., Garret C., Kayla G., Kyle R., Samuel R., Magdalena B., Gaetano B. (2009). Building the Internet of things using RFID: The RFID ecosystem experience. IEEE Internet Comp..

[2] Roy W. (2004). The Magic of RFID. ACM Queue.

[3] Fusheng W., Peiya L. Temporal Management of RFID Data.

[4] Sudarshan S.C., Venkat K., Sridhar R., Sanjay S. Managing RFID Data.

[5] Shawn R.J., Minos G., Michael J.F. Adaptive Cleaning for RFID Data Streams.

[6] Sheng Q., Zeadally S., Luo Z., Chung J.Y., Maamar Z. (2010). Ubiquitous RFID: Where are we?. Inf. Sys. Front..

[7] Mahdin H., Abawajy J. (2011). An approach for removing redundant data from RFID data streams. Sensors.

[8] Massawe L.V., Kinyua J.D.M., Vermaak H. (2012). Reducing false negative reads in RFID data streams using an adaptive sliding-window approach. Sensors.

[9] Bornhovd C., Haller S., Schaper J. Integrating Automatic Data Acquisition with Business Processes Experiences with SAP's Auto-ID Infrastructure.

[10] Lin D., Elmongui H., Bertino E., Ooi B., Wagner R., Revell N., Pernul G. (2007). Data Management in RFID Applications. Database and Expert Systems Applications.

[11] Lee C.H. (2011). RFID data processing in supply chain management using a path encoding scheme. IEEE Trans. Knowl. Data Eng..

[12] Shawn R.J., Michael J.F., Minos G. (2008). An adaptive RFID middleware for supporting metaphysical data independence. VLDB J..

[13] Jun R., Sangeeta D., Hetal T., Latha S.C. A Deferred Cleansing Method for RFID Data Analytics.

[14] Chen H., Ku W.S., Wang H., Sun M.T. (2010). Leveraging Spatio-Temporal Redundancy for RFID Data Cleansing.

[15] Gonzalez H., Han J., Shen X. Cost-Conscious Cleaning of Massive RFID Data Sets.

[16] Darcy P., Stantic B., Sattar A. A Fusion of Data Analysis and Non-Monotonic Reasoning to Restore Missed RFID Readings.

